# Author Correction: Mutant p53 sustains serine-glycine synthesis and essential amino acids intake promoting breast cancer growth

**DOI:** 10.1038/s41467-023-43018-3

**Published:** 2023-11-06

**Authors:** Camilla Tombari, Alessandro Zannini, Rebecca Bertolio, Silvia Pedretti, Matteo Audano, Luca Triboli, Valeria Cancila, Davide Vacca, Manuel Caputo, Sara Donzelli, Ilenia Segatto, Simone Vodret, Silvano Piazza, Alessandra Rustighi, Fiamma Mantovani, Barbara Belletti, Gustavo Baldassarre, Giovanni Blandino, Claudio Tripodo, Silvio Bicciato, Nico Mitro, Giannino Del Sal

**Affiliations:** 1 Department of Life Sciences, University of Trieste, 34127 Trieste, Italy; 2 International Centre for Genetic Engineering and Biotechnology (ICGEB), Area Science Park-Padriciano, 34149 Trieste, Italy; 3 DiSFeB, Dipartimento di Scienze Farmacologiche e Biomolecolari, University of Milan, Milan, Italy; 4 Tumor Immunology Unit, Department of Health Science, Human Pathology Section, School of Medicine, University of Palermo, 90133 Palermo, Italy; 5 Translational Oncology Research Unit, IRCCS Regina Elena National Cancer Institute, Rome, Italy; 6 Unit of Molecular Oncology, Centro di Riferimento Oncologico di Aviano (CRO), IRCCS, National Cancer Institute, 33081 Aviano, Italy; 7 IFOM ETS, the AIRC Institute of Molecular Oncology, Milan, Italy; 8 Center for Genome Research, University of Modena and Reggio Emilia, 41125 Modena, Italy; 9 Department of Experimental Oncology, IEO, European Institute of Oncology IRCCS, Milan, Italy

**Keywords:** Breast cancer, Cancer metabolism

Correction to: *Nature Communications* 10.1038/s41467-023-42458-1, published online 25 October 2023

In this article, the author name Ilenia Segatto was incorrectly written as Ilaria Segatto. The original article has been corrected.

