# Supplementary material for: Mutant p53 sustains serine-glycine synthesis and essential amino acids intake promoting breast cancer growth
Source: Nat Commun. 2023 Oct 25;14:6777. doi: 10.1038/s41467-023-42458-1 (PMC10600207; doi:10.1038/s41467-023-42458-1)
Supplement: Supplementary file 1 — Supplementary information [file 41467_2023_42458_MOESM1_ESM.pdf]

# SUPPLEMENTARY INFORMATION Tombari et al.

**Mutant p53 sustains serine-glycine synthesis and essential amino acids intake promoting breast cancer growth**

# SUPPLEMENTARY FIGURE 1

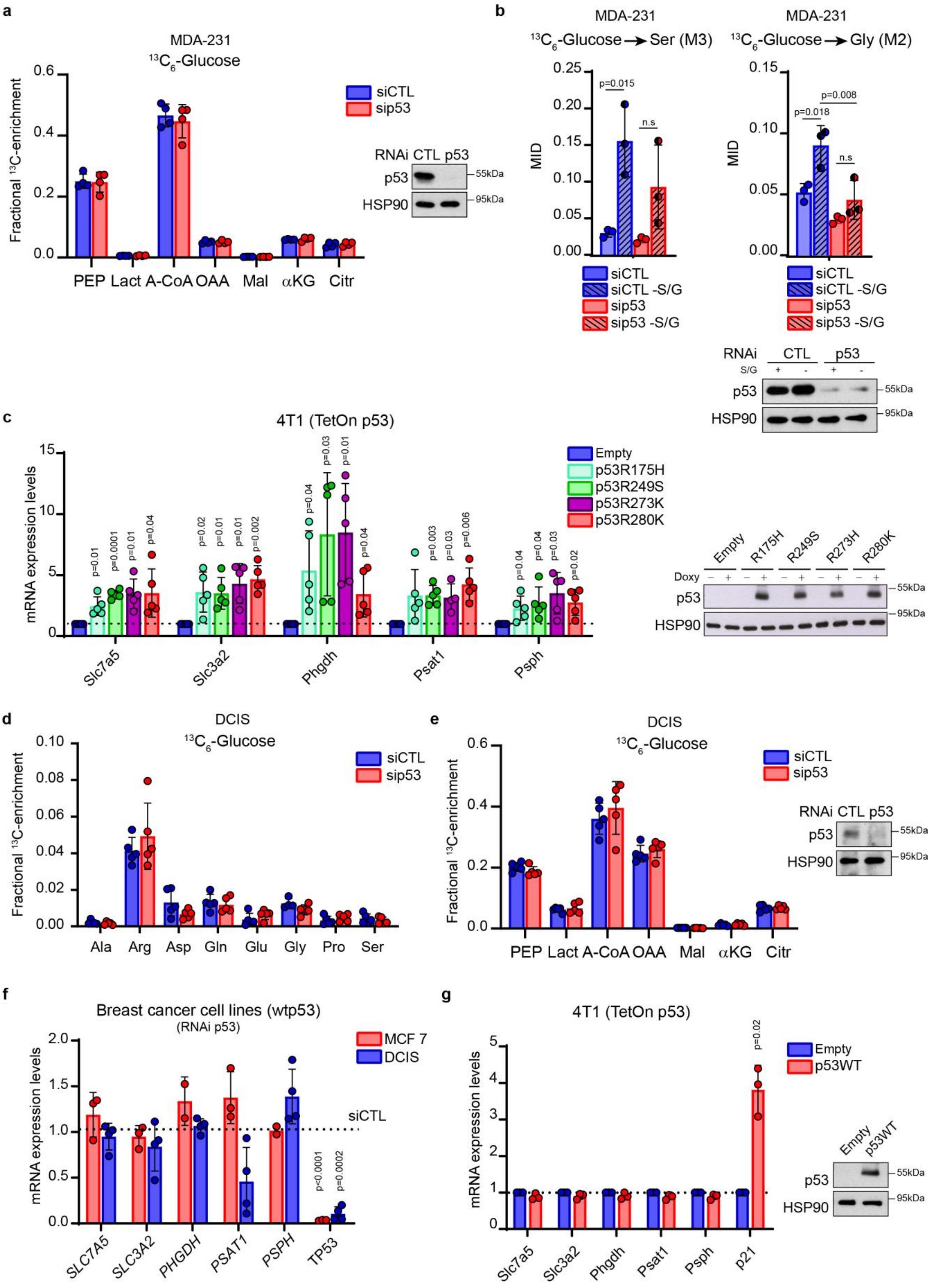

**Supplementary Figure 1**

**a.** Left panel: Mass isotopomer distribution (MID) of glycolysis and TCA cycle metabolites derived from [U-$^{13}C_6$]-Glucose in MDA-MB 231 cells upon control (siCTL) or p53 (sip53) silencing (PEP M3, Lact M3, A-CoA M2, OAA M4, Mal M2, αKG M5, Citr M6). For labeling experiments, cells were transfected with control or p53 siRNAs on the day of seeding and cultivated for 24h followed by incubation with [U-$^{13}C_6$]-Glucose in the medium for additional 24h. Right panel: western blot analysis of p53 levels in the above-described condition. HSP90 was used as loading control; n=4 biological replicates.

**b**. Upper panels: mass isotopomer distribution (MID) of serine M3 (left) and glycine M2 (right) from labeled [U-$^{13}C_6$]-Glucose in MDA-MB 231 cells upon control (siCTL) or p53 (sip53) silencing in complete medium or medium without serine and glycine (-S/G). For labeling experiments, cells were transfected with control or p53 siRNAs the day of seeding followed by incubation with [U-$^{13}C_6$]-Glucose in complete medium or medium without serine and glycine (-S/G), both with dialyzed serum, for additional 24h. Lower panel: western blot analysis of p53 levels in the above-described condition. HSP90 was used as loading control; n=3 biological replicates.

**c.** Left panel: qRT-PCR analysis of expression of indicated genes in 4T1 TetOn inducible clones in presence of doxycycline 1µg/mL for 24h. mRNA levels relative to those measured in 4T1 clone expressing empty vector (dotted line) are shown. Right panel: western blot analysis of p53 levels in 4T1 TetOn inducible clones in presence (+) or absence (-) of doxycycline 1µg/mL for 24h; n=5 biological replicates.

**d.** Mass isotopomer distribution (MID) of AAs from [U-$^{13}C_6$]-Glucose in MCF10DCIS.com cells upon control (siCTL) or p53 (sip53) silencing (isotopomers displayed in Fig. 1a are those reported in the graph). For labeling experiments, cells were transfected with control or p53 siRNAs on the day of seeding and cultivated for 24h followed by incubation with [U-$^{13}C_6$]-Glucose in the medium for additional 24h; n=5 biological replicates.

**e.** Left panel: Mass isotopomer distribution (MID) of glycolysis and TCA cycle metabolites derived from [U-$^{13}C_6$]-glucose in MCF10DCIS.com cells upon the above-described conditions (PEP M3, Lact M3, A-CoA M2, OAA M4, Mal M2 αKG M5, Citr M6). Right panel: western blot analysis of p53 levels in the above-described condition. HSP90 was used as loading control; n=5 biological replicates.

**f.** qRT-PCR analysis of the indicated genes in MCF7 and MCF10DCIS.com cell lines upon silencing of p53. mRNA expression levels relative to those measured in control condition (dotted line) are shown; n=3 biological replicates for MCF7 and n=4 biological replicates for MMCF10DCIS.com.

**g.** Left panel: qRT-PCR analysis of expression of indicated genes in 4T1 TetOn inducible clones in presence of doxycycline 1μg/mL for 24h. mRNA levels relative to those measured in 4T1 clone expressing empty vector (dotted line) are shown. Right panel: western blot analysis of p53 levels in 4T1 TetOn inducible clones in presence of doxycycline 1μg/mL for 24h; n=5 biological replicates.

Graph bars represent mean ± s.d. Two-tailed Student's t-test or one-way ANOVA (Fisher's LSD).

# SUPPLEMENTARY FIGURE 2

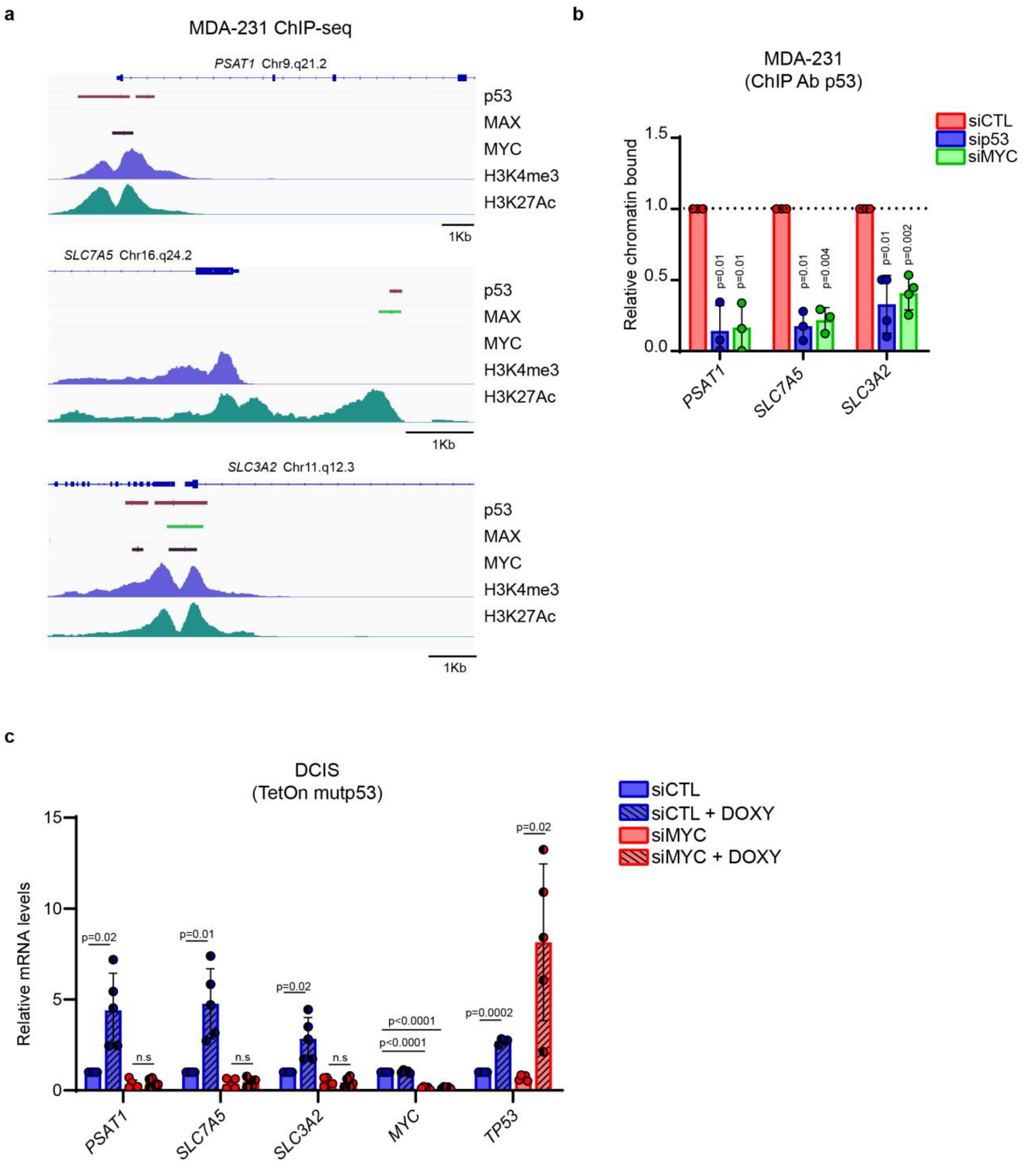

**Supplementary Figure 2**

**a.** Scheme of *PSAT1*, *SLC7A5*, and *SLC3A2* loci, indicating promoter marks (H3K4me3 and H3K27Ac), putative TSS, and regions bound by mutp53, MAX and MYC.

**b.** Chromatin immunoprecipitation (ChIP) analysis of MDA-MB-231 cells upon control (siCTL), p53 (sip53) or MYC (siMYC) silencing with anti-p53 DO-1 antibody. Specific p53 binding to the indicated promoters was calculated as the ratio of fraction of input chromatin bound (2-ΔCT method) in p53-silenced (sip53) and MYC-silenced (siMYC) vs control-silenced (siCTL) cells. n=3 biological replicates for *PSAT1*, n=3 biological replicates for *SLC7A5*, n=4 biological replicates for *SLC3A2.*

**c.** qRT-PCR analysis of indicated genes in MCF10DCIS.COM TetOn inducible clones cultured upon control (siCTL) or MYC (siMYC) silencing in presence of doxycycline 1μg/mL for 3 days; n=5 biological replicates.

Graph bars represent mean ± s.d. Two-tailed Student's t-test.

## SUPPLEMENTARY FIGURE 3

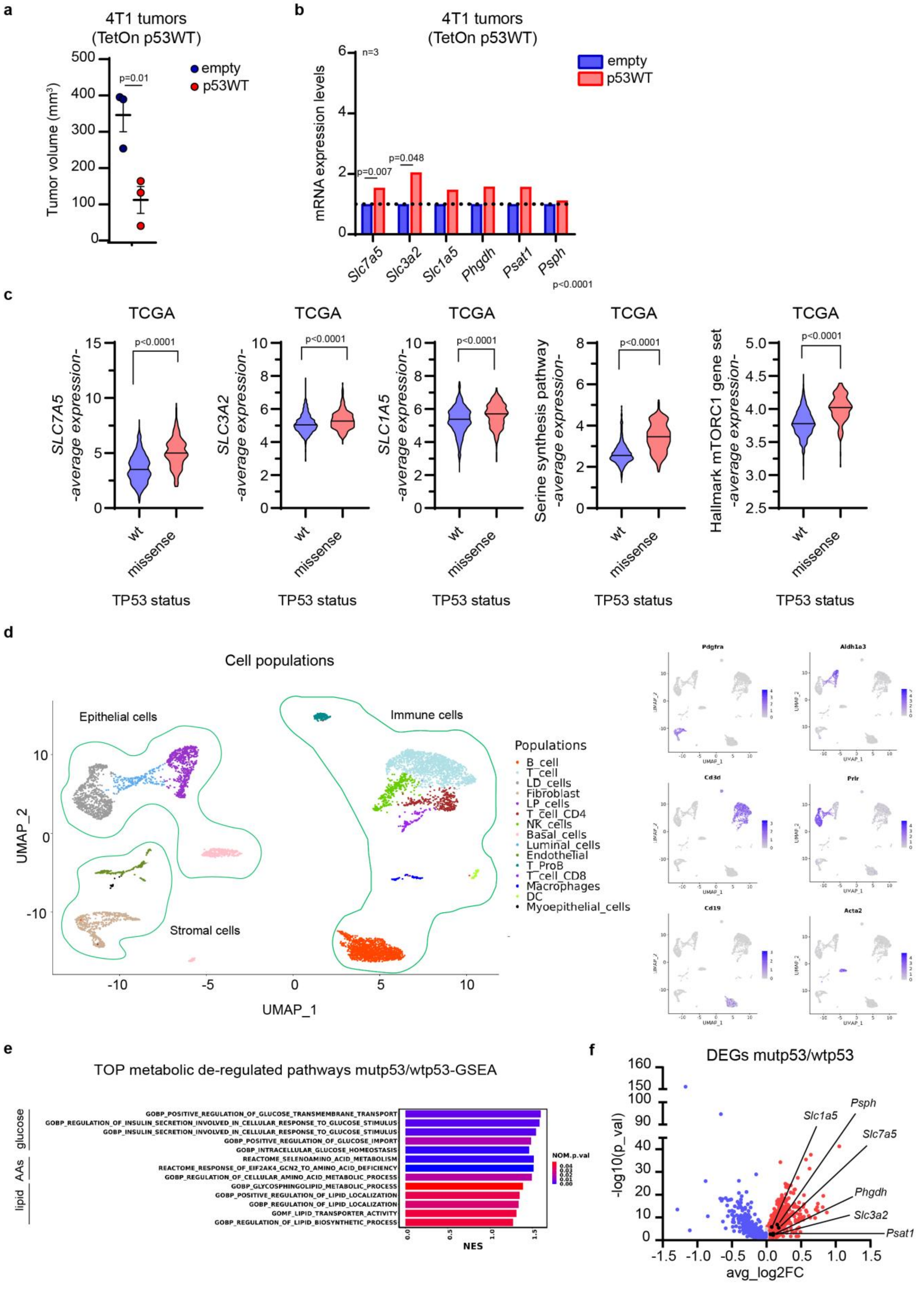

**Supplementary Figure 3**

**a.** Quantification of tumor volume in mice injected with indicated 4T1 TetOn inducible clones at day 24; n=3/condition.

**b.** qRT-PCR analysis of the indicated genes in 4T1 TetOn clones used in (**a)** n=3/condition.

**c.** Average expression levels of *SLC7A5*, *SLC3A2*, *SLC1A5*, serine synthesis pathway genes (i.e., *PHGDH*, *PSAT1*, and *PSPH*), and a gene set of mTORC1 activation in human breast cancer samples of the TCGA dataset (n=701) classified according to p53 status (wt and missense TP53 mutations).

**d.** Left panel: Uniform Manifold Approximation and Projection (UMAP) representation of scRNA-seq from wtp53 and mutp53 mice samples (wtp53: 3169 cells; mutp53: 3382 cells) colored according to cell type. Clusters were grouped into epithelial, stromal and immune populations. Right panels: FeaturePlots of known specific marker genes of the six more represented cell populations.

Abbreviations: LP, luminal progenitors; LD, luminal differentiated; DC, dendritic cells; NK, natural killer cells.

**e.** Gene sets enrichment analysis (GSEA) of glucose, AAs and lipid metabolism significantly enriched (NOM.p.val < 0.05) in mammary epithelial cells from $p53^{R172H/R172H}$ vs $p53^{+/+}$ mouse. The positive normalized enrichment score (NES) indicates the degree to which gene sets are overrepresented in the above-described conditions.

**f.** Volcano plot of genes significantly upregulated (red dots) and downregulated (blue dots) (padj<0.05) in mammary epithelial cells from $p53^{R172H/R172H}$ vs $p53^{+/+}$ mouse.

Two-tailed Student's t-test or Weighted Kolmogorov–Smirnov-like statistic or Wilcoxon Rank Sum test (pval) and bonferroni correction (padj).

# SUPPLEMENTARY FIGURE 4

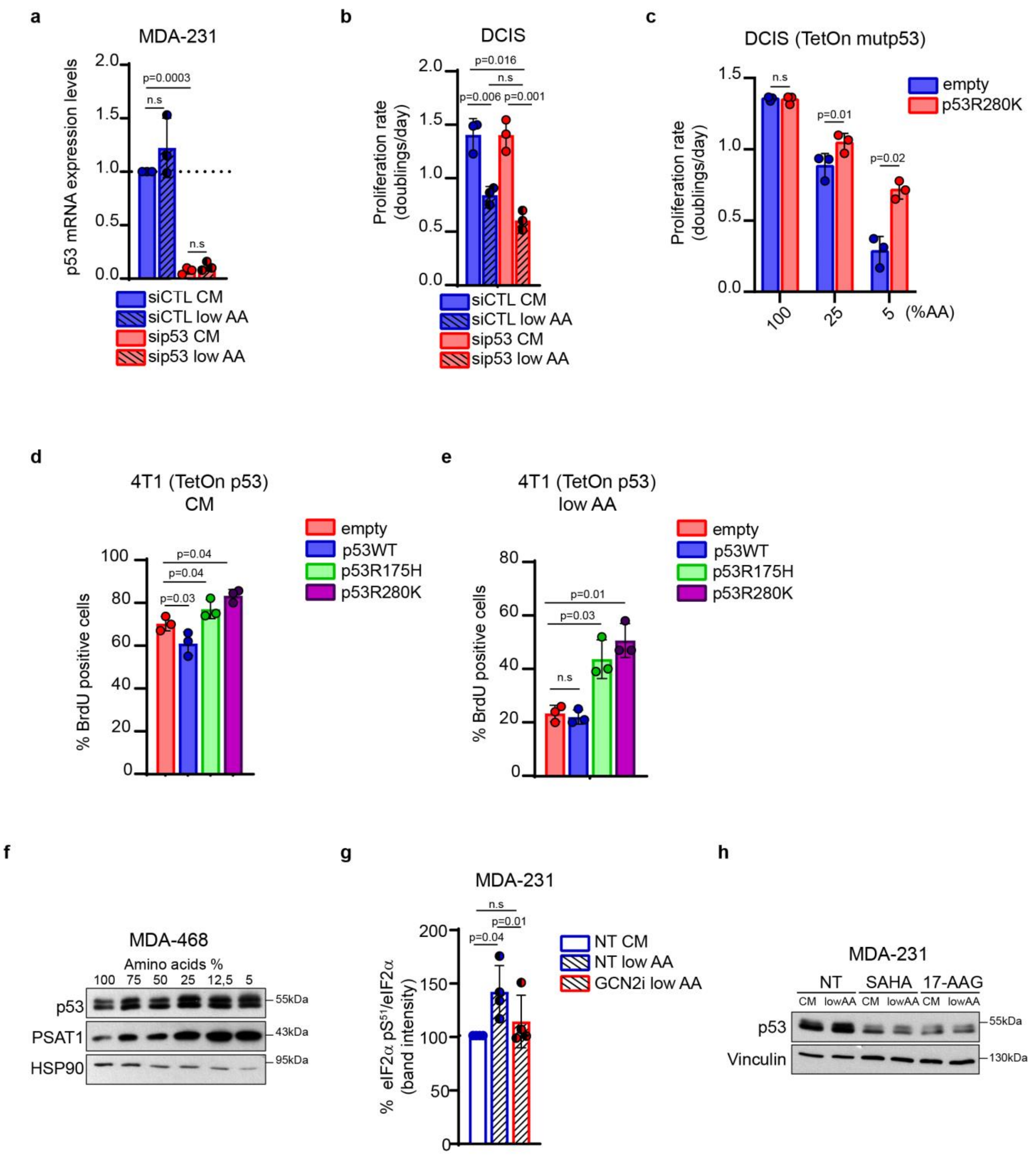

**Supplementary Figure 4**

**a.** qRT-PCR analysis of p53 mRNA level in MDA-MB 231cells grown for 72h in complete medium (CM) or medium containing 25% of AAs (low AA) upon silencing of p53. mRNA expression levels relative to those measured in control condition (dotted line) are shown; n=3 biological replicates.

**b.** Proliferation rate (doublings /day) of MCF10DCIS.COM cells cultured in complete medium (CM) or medium containing 25% of AAs (low AA) upon silencing of p53 for 3 days; n=3 biological replicates.

**c.** Proliferation rate (doublings/day) of MCF10DCIS.COM TetOn inducible clones cultured in medium with the indicated percentage of AAs for 3 days in presence of doxycycline 1μg/mL; n=3 biological replicates.

**d-e.** BrdU incorporation analysis in indicated 4T1 TetOn inducible clones grown in complete medium (CM) (**d**) or medium containing 25% of AAs (low AA) (**e**), in presence of doxycycline 1μg/mL. BrdU was added 3h (**d**) or 12h (**e**) before the end of the experiment. The percentage of BrdU positive cells out of 100-150 nuclei counted for each condition is shown; n=3 biological replicates.

**f.** Western blot analysis of the indicated proteins in MDA-MB 468 cells grown for 72h in the indicated percentage of AAs; n=3.

**g.** Quantification of western blot shown in Fig. 3f. The percentage of eIF2α pS51 relative to eIF2α is shown; n=4 biological replicates.

**h.** Western blot analysis of the indicated proteins in MDA-MB 231 cells grown for 72h in complete medium (CM) or medium containing 25% of AAs (low AA), upon treatment with DMSO (NT), SAHA 1 μM, and 17-AAG 5 μM for 48h. Vinculin was used as loading control; n=3.

Graph bars represent mean ± s.d.. Two-tailed Student's t-test.

# SUPPLEMENTARY FIGURE 5

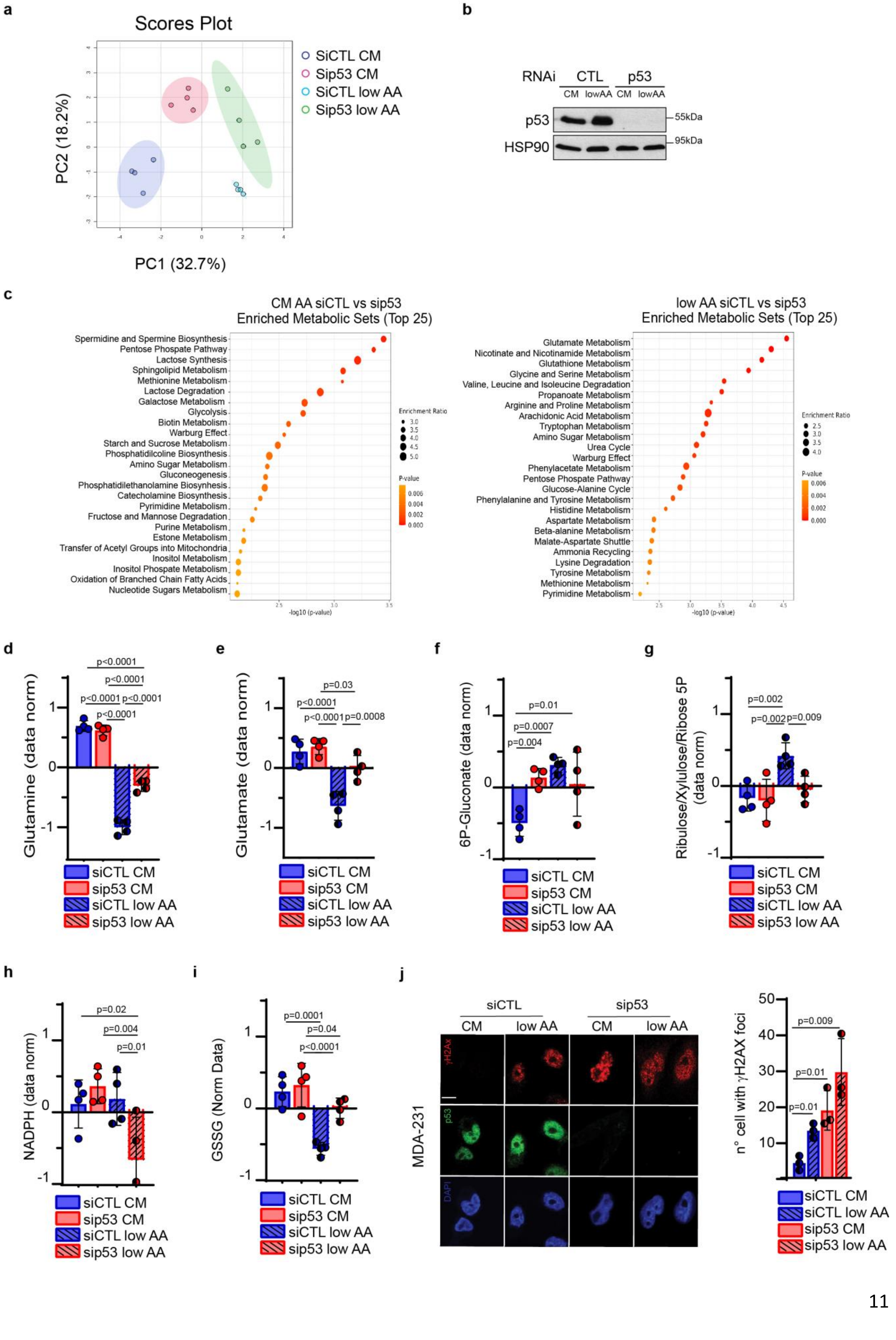

**Supplementary Figure 5**

**a.** Principal component analysis (PCA) plot based on the metabolomic profile of MDA-MB 231 cells cultured for 3 days in complete medium (CM) or medium containing 25% of AAs (low AA) upon control (siCTL) or p53 (sip53) silencing. n=4 independent replicates (one single experiment).

**b.** Western blot analysis of p53 levels in the above-described conditions. HSP90 was used as loading control.

**c.** Left panel: dot Plot of the top 25 Enriched Metabolites Sets - quantitative enrichment analysis (Pathway based – SMPDB) on the results of metabolomic analysis (FDR < 0.05) in MDA-MB-231 cells cultured in control silencing (siCTL) vs p53 silencing (sip53) in complete medium (CM). Right panel: dot Plot of the top 25 Enriched Metabolites Sets - quantitative enrichment analysis (Pathway based – SMPDB) on the results of metabolomic analysis (FDR < 0.05) in MDA-MB-231 cells cultured in control silencing (siCTL) vs p53 silencing (sip53) in medium containing 25% of AAs (low AA). n=4 independent replicates (one single experiment).

**d-i**. Histograms showing the normalized abundance of glutamine (**d**), glutamate (**e**), 6-phosphogluconate (**f**), ribose-5-phosphate/xylulose-5-phosphate/ribulose-5-phosphate (three different isomers here measured as sum) (**g**), NADPH (**h**) and oxidized glutathione (i) from LC-MS analysis. n=4 independent replicates (one single experiment).

**j.** Left panel: representative immunofluorescence images of γH2AX positive MDA-MB 231 cells, cultured for 3 days in complete medium (CM) or in medium containing 25% of AAs (low AA) upon control (siCTL) or p53 (sip53) silencing. Right panel: quantification of γH2AX positive MDA-MB 231 cells. Scale bar 20 μm. The number of γH2AX positive cells out of 100 cells/condition is shown; n=3 biological replicates.

Two-tailed Student’s t-test or Ordinary one-way ANOVA test (Fisher’s LSD).

# SUPPLEMENTARY FIGURE 6

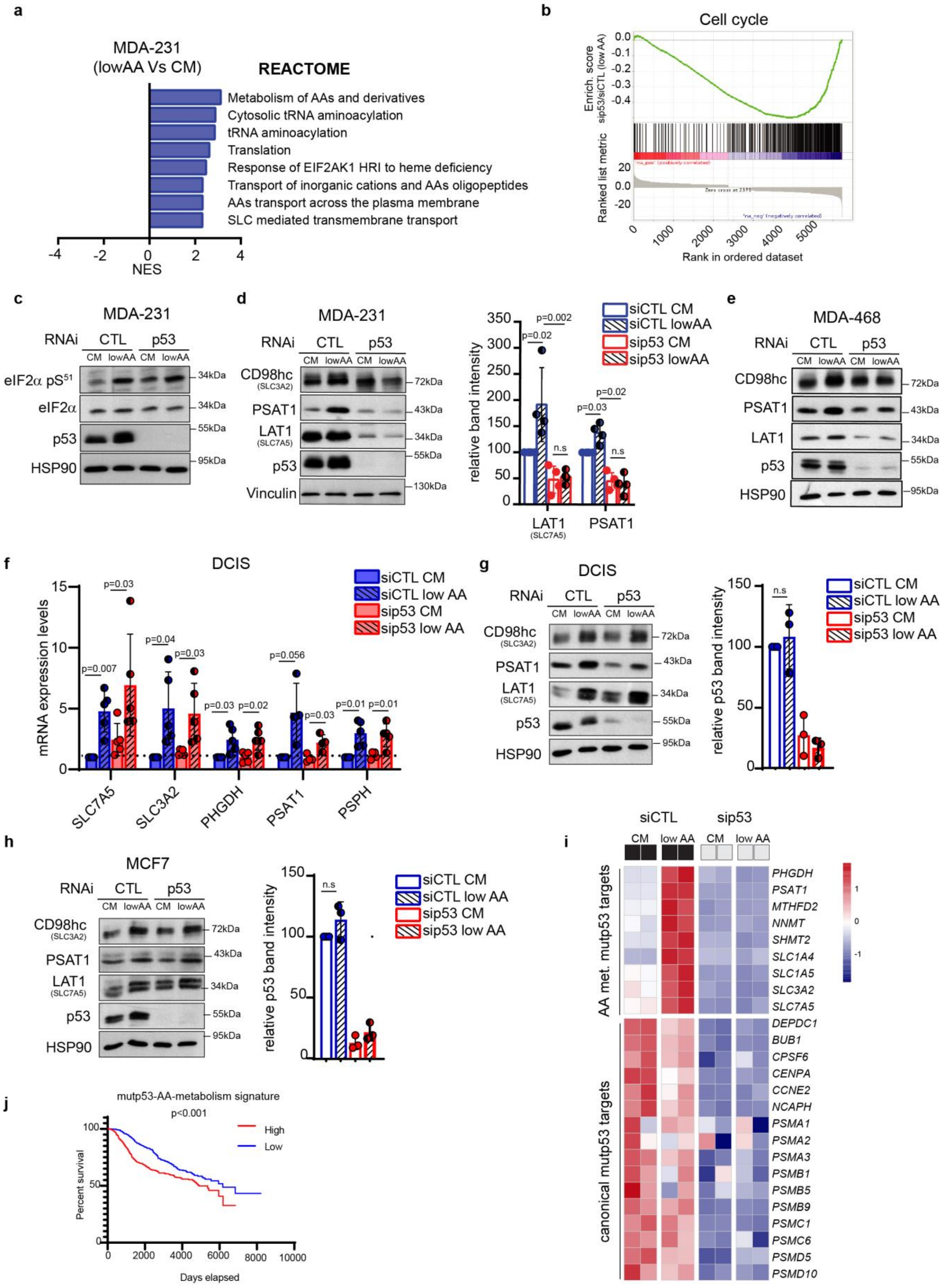

**Supplementary Figure 6**

**a.** Reactome gene sets significantly enriched (padj value < 0.05) in MDA-MB-231 cells cultured in low AA vs complete medium (CM) in control silencing. The positive normalized enrichment score (NES) indicates the degree to which Reactome gene sets are overrepresented in the conditions described above. Gene expression data were obtained from n=2 biological replicates for each condition (GSE214494).

**b.** Gene set enrichment analysis (GSEA) of “Cell cycle” in MDA-MB-231 cells cultured in low AA upon p53 silencing vs control silencing (sip53/siCTL). n=2 biological replicates for each condition (GSE214494).

**c, d.** Western blot analyses of the indicated proteins in MDA-MB 231 cells cultivated for 72h in complete medium (CM) and in medium containing 25% of AAs (low AA) upon silencing of p53 (**c**, **d** left panel). Quantification of LAT1 and PSAT1 western blot bands expressed as percentage relative to Vinculin. HSP90 and Vinculin were shown as loading controls (**d**, right panel); n=4 biological replicates.

**e.** Western blot analysis of the indicated proteins in MDA-MB 468 cells cultivated for 72h in complete medium (CM) and in medium containing 25% of AAs (low AA) upon silencing of p53. HSP90 was shown as loading control; n=3.

**f.** qRT-PCR of indicated genes in DCIS cells cultivated for 72h in complete medium (CM) or medium containing 25% of AAs (low AA) upon silencing of p53. mRNA levels relative to control condition (dotted line) are shown; n=5 biological replicates.

**g, h.** Left panels: western blot analysis of the indicated proteins in DCIS (**g**) and MCF7 (**h**) cells cultivated for 72h in complete medium (CM) and in medium containing 25% of AAs (low AA) upon silencing of p53. HSP90 was shown as loading control. Right panels: quantification of p53 levels relative to HSP90 in western blots; n=3.

**i.** Heatmap of RNA-seq data of genes related to AA biosynthesis, intake, and metabolism in MDA-MB 231 cells in complete medium (CM) and in medium containing 25% of AAs (low AA) upon

control (siCTL) or p53 (sip53) silencing. Two columns for each condition represent n=2 biological replicates.

**j.** Cumulative incidence of overall survival calculated using Kaplan-Meier survival analysis in human breast cancer samples of the Metabric dataset (n=997) stratified based on high or low mutp53 AA metabolism signature.

Graph bars represent mean ± s.d. Two-tailed Student's t-test or log-rank test or Wilcoxon Rank Sum test (pval) and bonferroni correction (padj).

# SUPPLEMENTARY FIGURE 7

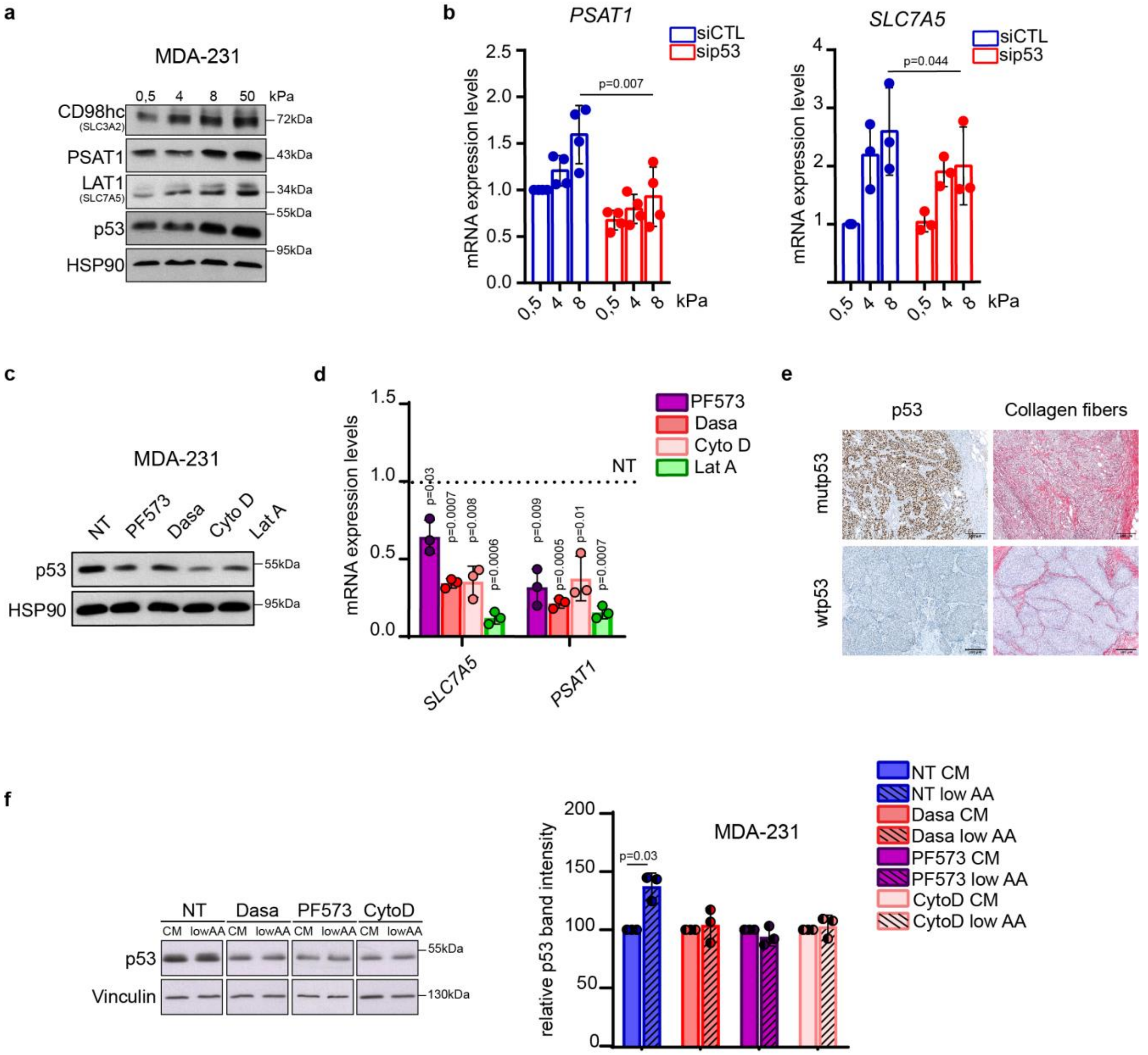

**Supplementary Figure 7.**

**a.** Western blot analysis of the indicated proteins in MDA-MB 231 cells cultured on fibronectin-coated hydrogels with 0,5, 4, 8, and 50 kPa elastic moduli for 48h. HSP90 was used as loading control; n=3.

**b.** qRT-PCR of indicated genes in MDA-MB 231 cells cultured on fibronectin-coated hydrogels with 0,5, 4 and 8 kPa elastic moduli for 48h upon control (siCTL) or p53 (sip53) silencing. n=4 biological replicates for *PSAT1* and n=3 biological replicates for *SLC7A5*.

**c.** Western blot analysis of p53 protein in MDA-MB 231 treated with DMSO (NT), PF573228 (PF573) 10 µM, Dasatinib (Dasa) 0,5 µM, Cytochalasin D (Cyto D) 1 µM, Latrunculin A (Lat A) 0,5 µM, for 48h. HSP90 was shown as loading control; n=3.

**d.** qRT-PCR of indicated genes in MDA-MB 231 cells treated with DMSO (NT), PF573228 (PF573) 10 µM, Dasatinib (Dasa) 0,5 µM, Cytochalasin D (Cyto D) 1 µM, Latrunculin A (Lat A) 0,5 µM, for 48h; n=3 biological replicates.

**e.** Representative images of p53 immunohistochemical staining and Picrosirius Red of breast cancer samples of Fig. 2d. Original magnification, x50. Scale bar, 500 µm.

**f.** Left panel: western blot analysis of p53 protein in MDA-MB 231 cultivated for 72h in complete medium (CM) and in medium containing 25% of AAs (low AA) and treated with DMSO (NT), Dasatinib (Dasa) 0,5µM, PF573228 (PF573) 10µM, and Cytochalasin D (Cyto D) 1µM. Vinculin was shown as loading control. Right panel: quantification of p53 levels relative to vinculin in western blot; n=3 biological replicates.

Graph bars represent mean ± s.d. Two-tailed Student's t-test.

# SUPPLEMENTARY FIGURE 8

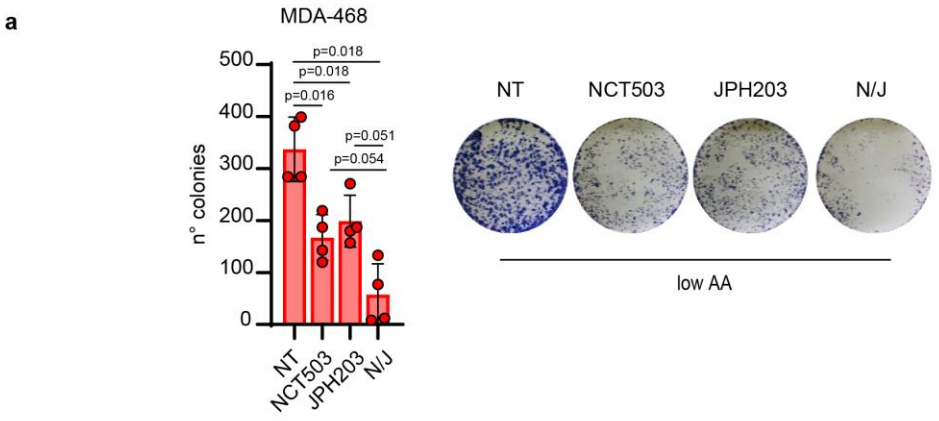


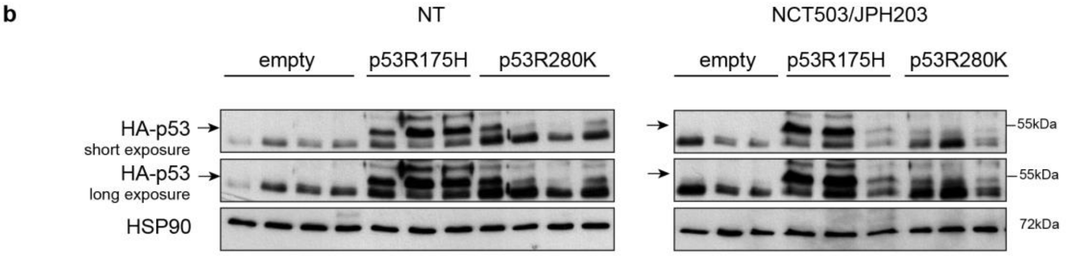


c

| | | gr (T0) | gr (T12) | gr (T24) |
|---|---|---|---|---|
| NT | empty | 21.6 | 21.9 | 22.4 |
| NT | empty | 21.8 | 22.3 | 22.9 |
| NT | empty | 23.3 | 24 | 24.5 |
| NT | empty | 20.4 | 21.1 | 22.4 |
| NT | p53R280K | 21 | 21.6 | 22 |
| NT | p53R280K | 20.7 | 21 | 21.6 |
| NT | p53R280K | 24 | 24.7 | 25.7 |
| NT | p53R280K | 24 | 24.5 | 25 |
| NT | p53R175H | 21.3 | 21.8 | 22.4 |
| NT | p53R175H | 16.2 | 17.1 | 18.3 |
| NT | p53R175H | 21.5 | 22.2 | 23.1 |
| NCT503/JPH203 | empty | 20.3 | 21 | 22.5 |
| NCT503/JPH203 | empty | 21.4 | 22.1 | 23.2 |
| NCT503/JPH203 | empty | 21.4 | 21.8 | 22.9 |
| NCT503/JPH203 | p53R280K | 22 | 22.6 | 23.5 |
| NCT503/JPH203 | p53R280K | 20.4 | 21 | 21.9 |
| NCT503/JPH203 | p53R280K | 21.8 | 22.5 | 23.9 |
| NCT503/JPH203 | p53R175H | 23.3 | 23.8 | 24.9 |
| NCT503/JPH203 | p53R175H | 22.5 | 23.1 | 24 |
| NCT503/JPH203 | p53R175H | 21 | 21.9 | 23.4 |

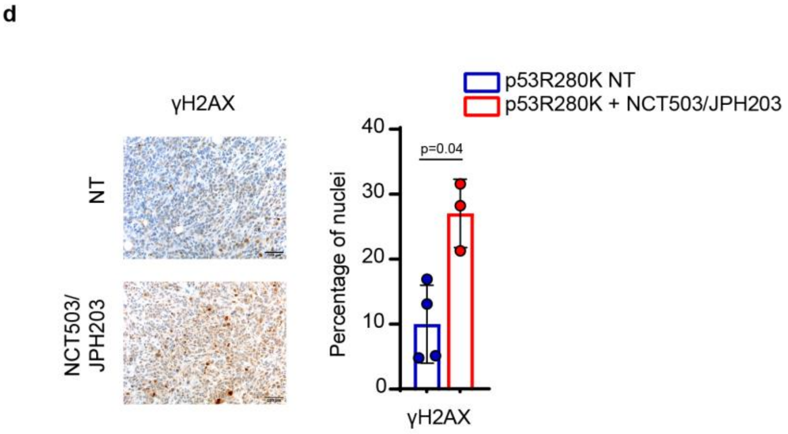


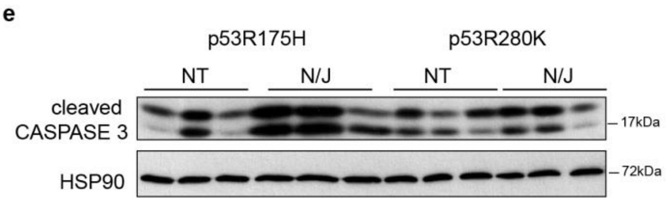

**Supplementary Figure 8**

**a.** Left panel: quantification of colonies formed by MDA-MB 468 cells grown in medium containing 25 % AAs (low AAs), treated with DMSO (NT), NCT-503 10μM, JPH203 10μM, or combination of NCT503 10μM - JPH203 10μM (N/J). Right panel: representative images of colonies described above; n=4 biological replicates.

**b.** Western blot analysis of HA-p53 protein in lysates of tumors formed by indicated 4T1 TetOn inducible clones treated with placebo (NT) or combination of NCT503/JPH203. HSP90 was shown as loading control.

**c**. Table indicating the body weight of mice at the day of injection (T0) and after 12 (T12) and 24 (T24) days.

**d.** Representative images and quantitative analyses of γH2AX immunohistochemical staining in mouse tumor samples of Fig. 6d and e. Original magnification, x200. Scale bar, 100 μm.

**e.** Western blot analysis of cleaved CASPASE 3 in lysates of tumors in **b**. HSP90 was shown as loading control.

Two-tailed Student's t-test.

# SUPPLEMENTARY FIGURE 9

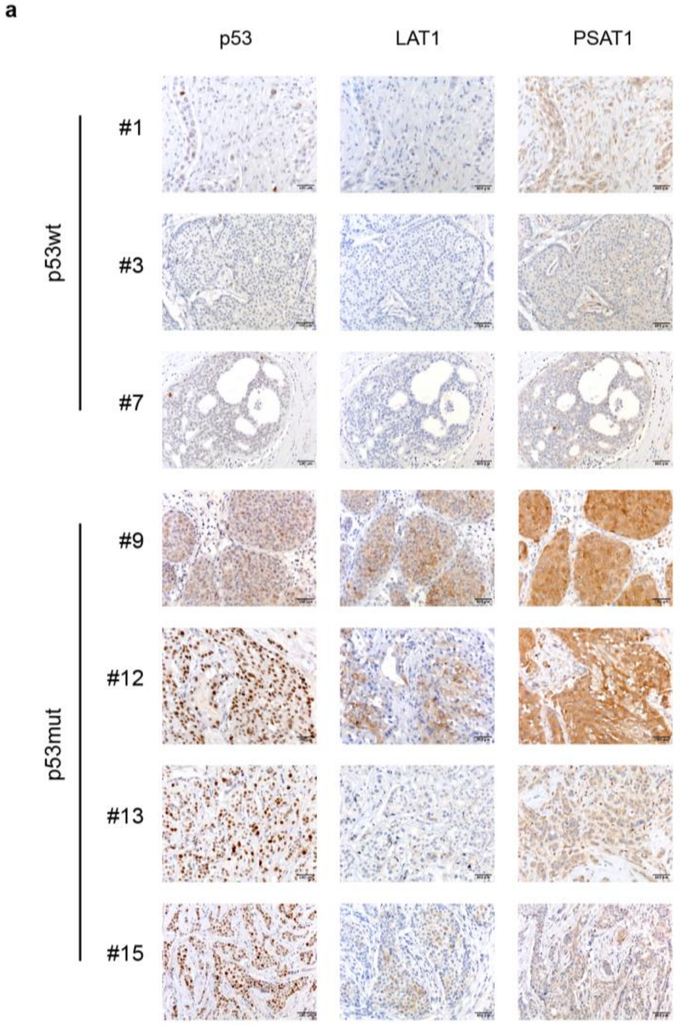


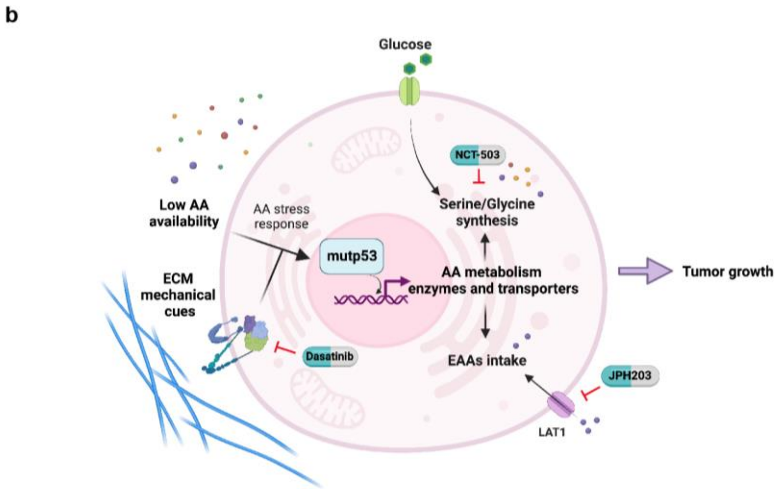

**Supplementary Figure 9**

**a.** Representative images of immunohistochemical staining of p53, PSAT1 and LAT1 in seven BCs cases from which PDOs were derived. Original magnification, x200. Scale bar, 100 µm.

**b.** Schematic summary model. Mutp53 potentiates two branches of amino acid metabolism (i.e., serine/glycine synthesis and EAAs intake) promoting the expression of SSP enzymes and EAAs transporter in response to environmental stresses to sustain tumor growth. Treating patient-derived tumor organoids bearing mutp53 with SSP and LAT1 inhibitors or with mechanosignaling inhibitor (e.g., Dasatinib) restricts their viability. Created with BioRender.com.

**Supplementary Table 1**: List of AA metabolism-related genes presenting at least one p53 peak in their promoter region in ChIP-seq of MDA-MB-231 cells (GEO GSE95303). The columns show gene name, peak number, peak score, peak genomic coordinates, histone marks (GEO GSE49651) and TFs bound.

| Gene Symbols | mutp53 Peak | Score | Chromosome | Peak Start | Peak End | Peak H3K27Ac Score | Peak H3K27Ac Start | Peak H3K27Ac End | Peak H3K4me3 Score | Peak H3K4me3 Start | Peak H3K4me3 End | TFs bound |
|---|---|---|---|---|---|---|---|---|---|---|---|---|
| AHCY | Peak1 | 98 | chr20 | 34312651 | 34313044 | 660,22 | 34311907 | 34317582 | - | - | - | |
| | Peak2 | 37 | chr20 | 34314324 | 34314819 | 660,22 | 34311907 | 34317582 | - | - | - | |
| | Peak3 | 19 | chr20 | 34316205 | 34316664 | 660,22 | 34311907 | 34317582 | - | - | - | |
| ASL | Peak1 | 3 | chr7 | 66075633 | 66075816 | 447,09 | 66074860 | 66077308 | 3100 | 66074899 | 66077182 | |
| | Peak2 | 3 | chr7 | 66075997 | 66076291 | 447,09 | 66074860 | 66077308 | 3100 | 66074899 | 66077182 | |
| ASS1 | Peak1 | 3 | chr9 | 130445623 | 130445792 | - | - | - | 2535,7 | 130444012 | 130446241 | |
| BCAT2 | Peak1 | 7 | chr19 | 48812180 | 48812653 | 414,47 | 48809919 | 48813326 | 3100 | 48809825 | 48813288 | |
| | Peak2 | 21 | chr19 | 48810720 | 48811297 | 414,47 | 48809919 | 48813326 | 3100 | 48809825 | 48813288 | |
| CTH | Peak1 | 11 | chr1 | 70410503 | 70411604 | 618,26 | 70409582 | 70412708 | 3109,63 | 70409883 | 70413167 | |
| DHFR | Peak1 | 10 | chr5 | 80655042 | 80655377 | 1920,97 | 80652883 | 80656825 | 3100 | 80652808 | 80656357 | |
| | Peak2 | 4 | chr5 | 80654707 | 80654865 | 1920,97 | 80652883 | 80656825 | 3100 | 80652808 | 80656357 | |
| | Peak3 | 4 | chr5 | 80654054 | 80654504 | 1920,97 | 80652883 | 80656825 | 3100 | 80652808 | 80656357 | |
| MAT2B | Peak1 | 11 | chr5 | 163503160 | 163503490 | 3100 | 163502173 | 163507918 | 3100 | 163502616 | 163507814 | |
| | Peak2 | 6 | chr5 | 163504630 | 163504939 | 3100 | 163502173 | 163507918 | 3100 | 163502616 | 163507814 | |
| | Peak3 | 4 | chr5 | 163505343 | 163505693 | 3100 | 163502173 | 163507918 | 3100 | 163502616 | 163507814 | |
| | Peak4 | 5 | chr5 | 163505819 | 163506017 | 3100 | 163502173 | 163507918 | 3100 | 163502616 | 163507814 | |
| MTHFD1 | Peak1 | 20 | chr14 | 64387787 | 64388716 | 2313,71 | 64386818 | 64390404 | 3100 | 64386741 | 64390029 | |
| MTHFD1L | Peak1 | 9 | chr6 | 150857628 | 150857864 | 713,62 | 150856301 | 150858872 | - | - | - | |
| | Peak2 | 10 | chr6 | 150865420 | 150865695 | 3100 | 150864600 | 150868032 | 3100 | 150864705 | 150867797 | |
| | Peak3 | 3 | chr6 | 150866826 | 150867053 | 3100 | 150864600 | 150868032 | 3100 | 150864705 | 150867797 | |
| MTHFD2 | Peak1 | 9 | chr2 | 74198239 | 74198814 | 884,91 | 74197934 | 74200401 | 3100 | 74197995 | 74200656 | |
| | Peak2 | 9 | chr2 | 74199081 | 74199427 | 884,91 | 74197934 | 74200401 | 3100 | 74197995 | 74200656 | |
| MTHFR | Peak1 | 27 | chr1 | 11805395 | 11806282 | 2793,98 | 11800238 | 11808449 | 1105,45 | 11804400 | 11807850 | |
| MTR | Peak1 | 31 | chr1 | 236794891 | 236795587 | 1498,38 | 236794048 | 236797389 | 3100 | 236794407 | 236797390 | |
| | Peak2 | 5 | chr1 | 236795660 | 236795860 | 1498,38 | 236794048 | 236797389 | 3100 | 236794407 | 236797390 | |
| MTRR | Peak1 | 15 | chr5 | 7868884 | 7869249 | 1144,86 | 7867900 | 7870481 | 3100 | 7867745 | 7870454 | |
| PHGDH | Peak1 | 5 | chr1 | 119711853 | 119712095 | - | - | - | 659,24 | 119711509 | 119713484 | |
| PSAT1 | Peak1 | 26 | chr9 | 78296255 | 78297415 | 3100 | 78295123 | 78299134 | 3100 | 78295572 | 78299291 | ZMYND8;BRD9;JUN;CDKN1B;BRD2;BRD4;SOX4;BRD2;ZMYND8;FOXM1;CDKN1B;ESR1;BRD9;JUN;FOSL1;MYC |
| | Peak2 | 7 | chr9 | 78297548 | 78297969 | 3100 | 78295123 | 78299134 | 3100 | 78295572 | 78299291 | no TFs |
| PSPH | Peak1 | 5 | chr7 | 56051790 | 56051948 | 2803,93 | 56049793 | 56054340 | 3100 | 56049924 | 56053245 | |
| | Peak2 | 16 | chr7 | 56051180 | 56051631 | 2803,93 | 56049793 | 56054340 | 3100 | 56049924 | 56053245 | |
| SHMT1 | Peak1 | 7 | chr17 | 18363438 | 18363605 | 444,51 | 18362019 | 18364398 | 3100 | 18361996 | 18364364 | |
| | Peak2 | 6 | chr17 | 18362713 | 18363247 | 444,51 | 18362019 | 18364398 | 3100 | 18361996 | 18364364 | |
| SLC1A5 | Peak1 | 43 | chr19 | 46787916 | 46789019 | 3100 | 46782271 | 46789346 | 3100 | 46783564 | 46789115 | |
| | Peak2 | 16 | chr19 | 46787125 | 46787503 | 3100 | 46782271 | 46789346 | 3100 | 46783564 | 46789115 | |
| SLC1A7 | Peak1 | 65 | chr1 | 53146101 | 53146747 | 669,13 | 53145095 | 53148852 | - | - | - | |
| SLC25A15 | Peak1 | 4 | chr13 | 40789811 | 40790206 | 957,39 | 40788391 | 40790805 | 3100 | 40788560 | 40790909 | |
| SLC38A2 | Peak1 | 9 | chr12 | 46373568 | 46374156 | 1581,38 | 46363889 | 46375053 | 3100 | 46366612 | 46374297 | |
| | Peak1 | 4 | chr12 | 46372993 | 46373342 | 1581,38 | 46363889 | 46375053 | 3100 | 46366612 | 46374297 | |
| | Peak1 | 17 | chr12 | 46372537 | 46372821 | 1581,38 | 46363889 | 46375053 | 3100 | 46366612 | 46374297 | |
| SLC3A2 | Peak1 | 6 | chr11 | 62854838 | 62855296 | 1633,76 | 62852395 | 62858111 | 3100 | 62852787 | 62858710 | BRD2, TP53, BRD4, MYC |
| | Peak2 | 70 | chr11 | 62855445 | 62856510 | 1633,76 | 62852395 | 62858111 | 3100 | 62852787 | 62858710 | BRD4;BRD2;MYC;BRD9;FOXM1;CDKN1B;SOX4;MAX;ZMYND8;TP53;YAP1;ESR1;TEAD4;JUN;FOSL1;STAT3 |
| SLC6A9 | Peak1 | 10 | chr1 | 44036619 | 44036876 | - | - | - | - | - | - | |
| | Peak2 | 9 | chr1 | 44031110 | 44031861 | 3162,8 | 44027570 | 44032401 | 3100 | 44028932 | 44032464 | |
| SLC7A1 | Peak1 | 13 | chr13 | 29594285 | 29594798 | 1929,22 | 29593831 | 29596880 | 3100 | 29593645 | 29596725 | |
| SLC7A5 | Peak1 | 14 | chr16 | 87871659 | 87871843 | 1126,78 | 87866285 | 87872163 | 3100 | 87866415 | 87869952 | JUN;YAP1;BRD4;TEAD4;E2F1;ZMYND8;CDKN1B;BRD2;BRD9;SOX4;STAT3;MAX;FOXM1 |
| SLC7A6 | Peak1 | 8 | chr16 | 68263906 | 68264329 | 3100 | 68263057 | 68267225 | 3100 | 68263308 | 68266840 | |
| | Peak2 | 13 | chr16 | 68265149 | 68265771 | 3100 | 68263057 | 68267225 | 3100 | 68263308 | 68266840 | |

**Supplementary Table 2:** List of known specific marker genes used to identify cell types of scRNAseq analysis of wtp53 mouse (p53$^{+/+}$) and mutp53 mouse (p53$^{R172H/R172H}$).

| Basal_cells | Luminal _cells | LD_cells | LP_cells | Myoepithelial _cells | Endothelial | Fibroblast | T_cell | CD4 | T_ProB | CD8 | B_cell | NK | Macrophage | DC |
|---|---|---|---|---|---|---|---|---|---|---|---|---|---|---|
| Trp63 | Epcam | Esr1 | Elf5 | Acta2 | Mcam | Vim | Cd3d | Cd4 | Il7r | Cd8a | Cd19 | Gnly | Cd80 | Cd1e |
| Snai2 | Krt18 | Pgr | Kit | Tagln | Vcam1 | Pdgfra | Cd3e | Ptprc | Ptprc | Ptprc | Cd22 | Nkg7 | Cd68 | Fcer1a |
| Krt14 | Krt8 | Ar | Cd14 | Mylk | Vwf | Pdgfrb | Tnfrsf4 | | | | Ptprc | Ptprc | C1qa | Cd208 |
| Krt5 | Krt19 | Foxa1 | Aldh1a3 | Myl9 | Pecam1 | | Ptprc | | | | | | C1qb | Cd265 |
| Itga6 | Gata3 | Prlr | Hey2 | Vim | Sele | | | | | | | | Adgre1 | Xcr1 |
| Procr | | Wnt4 | Ehf | | Cd93 | | | | | | | | Ptprc | Batf3 |
| Zeb2 | | Tnfsf11 | Tnfrsf11a | | Nectin3 | | | | | | | | | Fscn1 |
| | | Areg | | | Tek | | | | | | | | | |
| | | Cited1 | | | | | | | | | | | | |
| | | Batf | | | | | | | | | | | | |
| | | Tbx3 | | | | | | | | | | | | |

**Supplementary Table 3:** Percentages of cell populations obtained from scRNAseq analysis of wtp53 mouse ($p53^{+/+}$) and mutp53 mouse ($p53^{R172H/R172H}$).

| | **wtp53** | **mutp53** |
|---|---|---|
| B_cell | 21,7 | 17,3 |
| T_cell | 25,2 | 20,8 |
| LD_cells | 13,4 | 10,5 |
| Fibroblast | 5,7 | 9,7 |
| LP_cells | 8,5 | 10,9 |
| T_cell_CD4 | 4,9 | 5 |
| NK_cells | 4,7 | 5,1 |
| Basal_cells | 3,3 | 7,5 |
| Luminal_cells | 3 | 3,5 |
| Endothelial | 2,8 | 3,8 |
| T_ProB | 2 | 2 |
| T_cell_CD8 | 1,8 | 1,5 |
| Macrophages | 1,2 | 1,2 |
| DC | 1,5 | 0,8 |
| Myoepithelial_cells | 0,3 | 0,4 |

**Supplementary Table 4:** List of significant indicated DEGs from scRNAseq data of epithelial population comparing mutp53 mouse (p53$^{R172H/R172H}$) with wtp53 mouse (p53$^{+/+}$). DEGs have been calculated using the R package Seurat and sorted in decreasing avg_log2FC order. Wilcoxon Rank Sum test (pval) and bonferroni correction (padj). Cutoff padj<0.05.

| Gene | avg_log2FC | -log10(p_val) | p_val | pct.1 | pct.2 |
|---|---|---|---|---|---|
| Slc7a5 | 0,15 | 5,68 | 2,09E-06 | 0,491 | 0,369 |
| Psph | 0,12 | 6,84 | 1,45E-07 | 0,276 | 0,177 |
| Slc3a2 | 0,07 | 2,44 | 0,003666308 | 0,892 | 0,835 |
| Phgdh | 0,07 | 2,49 | 0,003216926 | 0,312 | 0,248 |
| Slc1a5 | 0,05 | 5,79 | 1,61E-06 | 0,298 | 0,201 |
| Psat1 | 0,04 | 2,53 | 0,002981782 | 0,386 | 0,31 |

**Supplementary Table 5:** List of 51 significantly different metabolites identified in the experimental conditions (SiQ 100%, Sip53 100%, SiQ 25%, Sip53 25%). ANOVA p-value (FDR) cutoff 0.05, post-hoc analysis Fisher's LSD.

| Metabolites | f.value | p.value | -LOG10(p) | FDR | Fisher's LSD |
|---|---|---|---|---|---|
| Glutamine | 260,14 | 3,51E-11 | 10,454 | 3,06E-09 | SiP53 100% - SiP53 25%; SiP53 100% - SiQ 25%; SiQ 100% - SiP53 25%; SiP53 25% - SiQ 25%; SiQ 100% - SiQ 25% |
| L-Acetylcarnitine | 65,21 | 1,07E-07 | 6,9724 | 4,64E-06 | SiP53 25% - SiP53 100%; SiQ 100% - SiP53 100%; SiQ 25% - SiP53 100%; SiP53 25% - SiQ 100%; SiQ 25% - SiQ 100% |
| Iso-Citrate | 51,536 | 3,97E-07 | 6,4017 | 1,15E-05 | SiP53 25% - SiP53 100%; SiQ 100% - SiP53 100%; SiQ 25% - SiP53 100%; SiQ 25% - SiQ 100% |
| pentanoylcarnitine | 35,106 | 3,21E-06 | 5,4938 | 6,98E-05 | SiP53 25% - SiP53 100%; SiP53 100% - SiQ 100%; SiP53 25% - SiQ 100%; SiP53 25% - SiQ 25%; SiQ 25% - SiQ 100% |
| Stearoylcarnitine | 33,497 | 4,12E-06 | 5,3852 | 7,17E-05 | SiP53 25% - SiP53 100%; SiP53 100% - SiQ 100%; SiP53 25% - SiQ 100%; SiP53 25% - SiQ 25%; SiQ 25% - SiQ 100% |
| Oleoylcarnitine | 28,035 | 1,05E-05 | 4,9787 | 0,0001372 | SiP53 100% - SiQ 100%; SiP53 100% - SiQ 25%; SiP53 25% - SiQ 100%; SiP53 25% - SiQ 25% |
| Malate | 27,767 | 1,10E-05 | 4,9571 | 0,0001372 | SiQ 100% - SiP53 100%; SiP53 100% - SiQ 25%; SiQ 100% - SiP53 25%; SiQ 100% - SiQ 25% |
| Putrescine | 24,799 | 1,98E-05 | 4,7042 | 0,00021492 | SiP53 25% - SiP53 100%; SiQ 100% - SiP53 100%; SiQ 25% - SiP53 100%; SiQ 100% - SiP53 25%; SiQ 25% - SiP53 25% |
| Glucose | 24,124 | 2,27E-05 | 4,6431 | 0,00021986 | SiP53 100% - SiQ 100%; SiQ 25% - SiP53 100%; SiP53 25% - SiQ 100%; SiQ 25% - SiQ 100% |
| Glutathione | 21,773 | 3,82E-05 | 4,4184 | 0,00033198 | SiP53 100% - SiP53 25%; SiQ 100% - SiP53 100%; SiP53 100% - SiQ 25%; SiQ 100% - SiP53 25%; SiP53 25% - SiQ 25%; SiQ 100% - SiQ 25% |
| Propionylcarnitine | 20,262 | 5,46E-05 | 4,263 | 0,00039695 | SiP53 25% - SiP53 100%; SiP53 100% - SiQ 100%; SiQ 25% - SiP53 100%; SiP53 25% - SiQ 100%; SiQ 25% - SiQ 100% |
| Asp | 19,995 | 5,83E-05 | 4,2347 | 0,00039695 | SiP53 100% - SiP53 25%; SiQ 100% - SiP53 100%; SiQ 100% - SiP53 25%; SiQ 25% - SiP53 25%; SiQ 100% - SiQ 25% |
| Glu | 19,922 | 5,93E-05 | 4,2268 | 0,00039695 | SiP53 100% - SiP53 25%; SiP53 100% - SiQ 25%; SiP53 25% - SiQ 25%; SiQ 100% - SiQ 25% |
| Fructose 1,6-bisphosphate | 18,428 | 8,69E-05 | 4,0612 | 0,00051977 | SiP53 25% - SiP53 100%; SiQ 100% - SiP53 100%; SiQ 25% - SiP53 100%; SiQ 100% - SiP53 25% |
| PEP | 18,16 | 9,33E-05 | 4,0303 | 0,00051977 | SiP53 25% - SiP53 100%; SiQ 25% - SiP53 100%; SiP53 25% - SiQ 100%; SiQ 25% - SiQ 100% |
| Fumarate | 18,067 | 9,56E-05 | 4,0196 | 0,00051977 | SiQ 100% - SiP53 100%; SiP53 100% - SiQ 25%; SiQ 100% - SiP53 25%; SiQ 100% - SiQ 25% |
| O-palmitoleoylcarnitine | 17,166 | 0,00012227 | 3,9127 | 0,00062573 | SiP53 100% - SiQ 100%; SiP53 100% - SiQ 25%; SiP53 25% - SiQ 100%; SiP53 25% - SiQ 25% |
| Thr | 16,385 | 0,00015267 | 3,8162 | 0,00073792 | SiP53 100% - SiP53 25%; SiP53 100% - SiQ 25%; SiQ 100% - SiP53 25%; SiQ 100% - SiQ 25% |
| GSSG | 15,475 | 0,00019984 | 3,6993 | 0,00091504 | SiP53 100% - SiP53 25%; SiP53 100% - SiQ 25%; SiP53 25% - SiQ 25%; SiQ 100% - SiQ 25% |
| L-Palmitoylcarnitine | 15,153 | 0,00022047 | 3,6567 | 0,00095903 | SiP53 25% - SiP53 100%; SiP53 100% - SiQ 100%; SiP53 25% - SiQ 100%; SiP53 25% - SiQ 25%; SiQ 25% - SiQ 100% |
| Ser | 13,935 | 0,00032453 | 3,4887 | 0,0013445 | SiP53 100% - SiP53 25%; SiP53 100% - SiQ 25%; SiQ 100% - SiP53 25%; SiQ 100% - SiQ 25% |
| Ile | 13,439 | 0,00038273 | 3,4171 | 0,0015135 | SiP53 100% - SiP53 25%; SiQ 100% - SiP53 25%; SiQ 25% - SiP53 25%; SiQ 100% - SiQ 25% |
| Phe | 12,98 | 0,00044771 | 3,349 | 0,0016935 | SiP53 100% - SiP53 25%; SiQ 100% - SiP53 25%; SiQ 25% - SiP53 25% |
| Met | 11,209 | 0,0008539 | 3,0686 | 0,0030012 | SiP53 100% - SiP53 25%; SiQ 100% - SiP53 25%; SiQ 25% - SiP53 25% |
| Citrate | 11,183 | 0,00086243 | 3,0643 | 0,0030012 | SiP53 25% - SiP53 100%; SiQ 100% - SiP53 100%; SiQ 25% - SiP53 100% |
| SAM | 10,806 | 0,00099927 | 3,0003 | 0,0033437 | SiQ 100% - SiP53 100%; SiQ 100% - SiP53 25%; SiQ 100% - SiQ 25% |
| UDP | 10,141 | 0,0013073 | 2,8836 | 0,0042124 | SiQ 100% - SiP53 100%; SiQ 100% - SiP53 25%; SiQ 100% - SiQ 25% |
| Lactate | 9,6655 | 0,0015959 | 2,797 | 0,0049588 | SiP53 25% - SiP53 100%; SiQ 100% - SiP53 100%; SiQ 25% - SiP53 100% |
| CTP | 9,5599 | 0,0016698 | 2,7773 | 0,0050094 | SiQ 100% - SiP53 100%; SiQ 100% - SiP53 25%; SiP53 25% - SiQ 25%; SiQ 100% - SiQ 25% |
| Asn | 9,4634 | 0,0017407 | 2,7593 | 0,0050482 | SiP53 100% - SiP53 25%; SiQ 100% - SiP53 25%; SiQ 25% - SiP53 25% |
| SAH | 9,2128 | 0,0019421 | 2,7117 | 0,0054505 | SiQ 100% - SiP53 100%; SiQ 25% - SiP53 100%; SiQ 100% - SiP53 25% |
| UTP | 8,8338 | 0,0023006 | 2,6382 | 0,0062548 | SiQ 100% - SiP53 100%; SiQ 100% - SiP53 25%; SiQ 100% - SiQ 25% |
| ATP | 8,5123 | 0,0026662 | 2,5741 | 0,0070292 | SiQ 100% - SiP53 100%; SiQ 100% - SiP53 25%; SiQ 100% - SiQ 25% |
| His | 8,3239 | 0,0029118 | 2,5358 | 0,0073529 | SiP53 100% - SiP53 25%; SiP53 100% - SiQ 25%; SiQ 100% - SiP53 25%; SiQ 100% - SiQ 25% |
| Val | 8,2905 | 0,0029581 | 2,529 | 0,0073529 | SiP53 100% - SiP53 25%; SiQ 100% - SiP53 25%; SiQ 25% - SiP53 25% |
| Dihydroxyacetone phosphate | 7,9143 | 0,0035428 | 2,4507 | 0,0085617 | SiQ 100% - SiP53 100%; SiQ 25% - SiP53 100%; SiQ 25% - SiP53 25% |
| Gly | 7,5897 | 0,0041575 | 2,3812 | 0,0097757 | SiP53 100% - SiP53 25%; SiP53 100% - SiQ 25%; SiQ 100% - SiP53 25%; SiQ 100% - SiQ 25% |
| Gluconate 6P | 7,4726 | 0,004409 | 2,3557 | 0,010094 | SiP53 100% - SiQ 100%; SiP53 25% - SiQ 100%; SiQ 25% - SiQ 100% |
| Glucose 6-Phosphate | 7,148 | 0,0052048 | 2,2836 | 0,011611 | SiQ 25% - SiP53 100%; SiP53 25% - SiQ 100%; SiQ 25% - SiQ 100% |
| Ribu-5P | 7,0681 | 0,0054257 | 2,2655 | 0,011801 | SiQ 25% - SiP53 100%; SiQ 25% - SiP53 25%; SiQ 25% - SiQ 100% |
| GMP | 6,8058 | 0,006231 | 2,2054 | 0,012983 | SiP53 25% - SiP53 100%; SiQ 100% - SiP53 100%; SiQ 25% - SiP53 100% |
| Isobutyryl-L-carnitine | 6,7947 | 0,0062679 | 2,2029 | 0,012983 | SiP53 100% - SiQ 25%; SiP53 25% - SiQ 25%; SiQ 100% - SiQ 25% |
| aKG | 5,6482 | 0,011947 | 1,9227 | 0,024172 | SiP53 25% - SiP53 100%; SiP53 25% - SiQ 100%; SiP53 25% - SiQ 25% |
| dGTP | 5,562 | 0,012577 | 1,9004 | 0,024868 | SiQ 100% - SiP53 100%; SiQ 100% - SiP53 25%; SiQ 100% - SiQ 25% |
| Sarcosine | 5,1917 | 0,015757 | 1,8025 | 0,029916 | SiP53 100% - SiP53 25%; SiQ 25% - SiP53 25%; SiQ 25% - SiQ 100% |
| Taurine | 5,1856 | 0,015818 | 1,8009 | 0,029916 | SiP53 100% - SiQ 100%; SiP53 25% - SiQ 100%; SiP53 25% - SiQ 25% |
| NADPH | 4,9985 | 0,017783 | 1,75 | 0,032918 | SiP53 100% - SiP53 25%; SiQ 100% - SiP53 25%; SiQ 25% - SiP53 25% |
| NADH | 4,9105 | 0,018806 | 1,7257 | 0,034086 | SiP53 25% - SiQ 100%; SiQ 25% - SiQ 100% |
| Ala | 4,8034 | 0,020144 | 1,6959 | 0,035766 | SiQ 25% - SiP53 25%; SiQ 25% - SiQ 100% |
| Ac-CoA | 4,6856 | 0,021743 | 1,6627 | 0,037833 | SiQ 100% - SiP53 25%; SiQ 25% - SiP53 25% |
| Arg | 4,3725 | 0,026765 | 1,5724 | 0,045658 | SiQ 25% - SiP53 25%; SiQ 25% - SiQ 100% |

**Supplementary Table 6**: Mutp53 AA metabolism signature. List of 213 genes found significantly upregulated in siCTL MDA-MB-231 cells grown in low AA vs CM condition, and downregulated in sip53 vs siCTL MDA-MB-231 cells grown in low AA condition.

| **mutp53 AA metabolism signature** | | | | | |
|---|---|---|---|---|---|
| TNFSF18 | EIF4EBP1 | GYPC | FADS3 | SLC43A1 | LDLRAP1 |
| PSAT1 | HIF1A-AS3 | SLFN12 | PSPH | LEPR | WWC3 |
| NXPH4 | PEAR1 | SLC1A3 | CHML | ROBO3 | FBXO25 |
| NNMT | ADM2 | LGALS8 | PDGFA | CCN2 | VLDLR |
| LINC02331 | ANOS1 | BEX2 | EPRS1 | RBCK1 | TRIB3 |
| ST6GALNAC3 | SHMT2 | AARS1 | SLC8B1 | TARS1 | LONP1 |
| SLC16A1 | ANKRD1 | PID1 | PHLDB3 | PYCR1 | RSL24D1 |
| JDP2 | NXN | NLRP1 | LINC00662 | ZNF598 | FLNB |
| APOL6 | ANKFN1 | CT83 | PCID2 | NARS1 | CHIC2 |
| GPR17 | SLC7A5 | VSIR | ACSS3 | RBMS2 | PRPF39 |
| PHGDH | CHAC1 | NCOA7 | CDC42EP1 | LMO4 | DDIT4 |
| PCK2 | NIBAN1 | OGFRL1 | NANOS1 | HSPA13 | LITAF |
| ASNS | OVCH2 | RPS6KA2 | LINC02693 | SPIRE1 | CALCOCO2 |
| SFTA1P | GPT2 | LINC02783 | TTC9 | UHRF1BP1 | SNTB1 |
| KISS1 | TLCD5 | RASGRF1 | TGFB2 | FAT3 | NTN4 |
| ANGPT4 | TPM1 | LIMCH1 | TIMM44 | NFE2L2 | CEP120 |
| TUBE1 | SARS1 | ATF3 | TMC8 | BCAT1 | MIR4435-2HG |
| SLC1A4 | MTHFD1L | SYTL1 | ANXA3 | PAWR | CEBPB |
| SHOC1 | SLC3A2 | WARS1 | SLC39A14 | DDX60L | ATF4 |
| FSTL1 | YARS1 | SLC7A1 | XPOT | GFPT1 | PPP1R15B |
| ASS1 | SLC22A15 | ADARB1 | MILR1 | UBE2J1 | FAM241A |
| VLDLR-AS1 | CASP4 | AXL | CARS1 | KLF13 | ADCY6 |
| C6orf132 | ELF3 | DOCK11 | CNGA1 | FMNL1-DT | TACC2 |
| FAM167A | TMEM156 | PDZD2 | LPAL2 | DYSF | ARHGEF17 |
| SLC1A5 | GARS1 | UGCG | EIF2S2 | PPARD | SLC38A1 |
| MTHFD2 | PABPC1L | SERTAD4 | HAX1 | PDLIM5 | KLHL42 |
| CSF2RB | TSEN15 | RPL13AP20 | SLC20A2 | SESN2 | RIOK3 |
| SORBS2 | PLXNA2 | PPL | CRIM1 | PRNP | PARP6 |
| MAP3K20 | PRKCE | MIR325HG | KLHL5 | IGDCC4 | WWC2 |
| CREB3L1 | AJUBA | MARS1 | ARHGEF2 | PXK | KIRREL1 |
| GABRG1 | CCND2 | CEBPG | ZNF419 | DNASE2 | DPY19L3 |
| PM20D2 | PPME1 | COL8A1 | ARHGAP23 | ECHDC2 | PARVA |
| CTH | PEA15 | MOCOS | LARS1 | LOC100128398 | SMIM13 |
| STC2 | PNMA2 | LSM8 | CLIC4 | TMEM268 | |
| MKX | SHANK2 | TOM1L1 | PDCD4 | CD55 | |
| INSYN2B | SLC7A11 | IARS1 | CHRM3-AS2 | ATP13A3 | |
