# Supplementary material for: Mutant p53 sustains serine-glycine synthesis and essential amino acids intake promoting breast cancer growth
Source: Nat Commun. 2023 Oct 25;14:6777. doi: 10.1038/s41467-023-42458-1 (PMC10600207; doi:10.1038/s41467-023-42458-1)
Supplement: Supplementary file 3 — Reporting Summary [file 41467_2023_42458_MOESM3_ESM.pdf]

nature portfolio

Corresponding author(s): Prof. Giannino Del Sal

Last updated by author(s): Oct 6, 2023

Reporting Summary

Nature Portfolio wishes to improve the reproducibility of the work that we publish. This form provides structure for consistency and transparency in reporting. For further information on Nature Portfolio policies, see our Editorial Policies and the Editorial Policy Checklist.

Statistics

For all statistical analyses, confirm that the following items are present in the figure legend, table legend, main text, or Methods section.

n/a	Confirmed	
☐	☒	The exact sample size (*n*) for each experimental group/condition, given as a discrete number and unit of measurement
☐	☒	A statement on whether measurements were taken from distinct samples or whether the same sample was measured repeatedly
☐	☒	The statistical test(s) used AND whether they are one- or two-sided<br>*Only common tests should be described solely by name; describe more complex techniques in the Methods section.*
☒	☐	A description of all covariates tested
☐	☒	A description of any assumptions or corrections, such as tests of normality and adjustment for multiple comparisons
☐	☒	A full description of the statistical parameters including central tendency (e.g. means) or other basic estimates (e.g. regression coefficient) AND variation (e.g. standard deviation) or associated estimates of uncertainty (e.g. confidence intervals)
☐	☒	For null hypothesis testing, the test statistic (e.g. *F*, *t*, *r*) with confidence intervals, effect sizes, degrees of freedom and *P* value noted<br>*Give P values as exact values whenever suitable.*
☒	☐	For Bayesian analysis, information on the choice of priors and Markov chain Monte Carlo settings
☒	☐	For hierarchical and complex designs, identification of the appropriate level for tests and full reporting of outcomes
☒	☐	Estimates of effect sizes (e.g. Cohen's *d*, Pearson's *r*), indicating how they were calculated

Our web collection on statistics for biologists contains articles on many of the points above.

Software and code

Policy information about availability of computer code

Data collection	Prism GraphPad 8 and Microsoft Office Excel 365.
Data analysis	Prism GraphPad, Microsoft Office Excel 365 and R 4.2.1 were used for all statistical analysis. For imaging analyses, Image J software from NIH and Adobe Photoshop CC 19 were used. For IHC imaging Nuclear Hub or Positive Pixel Count v9 ImageScope software were used. For bioinformatics analyses R 4.2.1 was used. For metabolic analyses, MetaboAnalyst 5.0 web tool was used.

For manuscripts utilizing custom algorithms or software that are central to the research but not yet described in published literature, software must be made available to editors and reviewers. We strongly encourage code deposition in a community repository (e.g. GitHub). See the Nature Portfolio guidelines for submitting code & software for further information.

Data

Policy information about availability of data

All manuscripts must include a data availability statement. This statement should provide the following information, where applicable:

- Accession codes, unique identifiers, or web links for publicly available datasets
- A description of any restrictions on data availability
- For clinical datasets or third party data, please ensure that the statement adheres to our policy

Data generated in this study are available upon request from the Corresponding author. The RNAseq datasets generated in this study have been deposited to GEO,

accession number GSE214494 (https://www.ncbi.nlm.nih.gov/geo/query/acc.cgi?acc=GSE214494). scRNAseq datasets generated during this study have been deposited to GEO, accession number GSE239706 (https://www.ncbi.nlm.nih.gov/geo/query/acc.cgi?acc=GSE239706).

Research involving human participants, their data, or biological material

Policy information about studies with human participants or human data. See also policy information about sex, gender (identity/presentation), and sexual orientation and race, ethnicity and racism.

Reporting on sex and gender	Female patients were recruited for the study. No other information was collected.
Reporting on race, ethnicity, or other socially relevant groupings	Patients recruited were female of all ages diagnosed for breast cancer. Then patients with annotated TP53 status were selected for generation of patient-derived-organoids.
Population characteristics	See above.
Recruitment	Patients were recruited according to breast cancer diagnosis.
Ethics oversight	Institutional review board of Regina Elena National Cancer Institute and Centro di Riferimento Oncologico di Aviano (CRO), National Cancer Institute and appropriate regulatory authorities (approval no. IFO 1270/19 and IRB-06-2017 respectively).

Note that full information on the approval of the study protocol must also be provided in the manuscript.

Field-specific reporting

Please select the one below that is the best fit for your research. If you are not sure, read the appropriate sections before making your selection.

☒ Life sciences ☐ Behavioural & social sciences ☐ Ecological, evolutionary & environmental sciences

For a reference copy of the document with all sections, see nature.com/documents/nr-reporting-summary-flat.pdf

Life sciences study design

All studies must disclose on these points even when the disclosure is negative.

Sample size	No statistical method was used to determine sample size. The sample size was chosen to include at least three biological replicates.
Data exclusions	No data were excluded from the analysis.
Replication	Experiments were performed succesfully at least three times. All replication attempts were succesfull.
Randomization	Animals were randomly put into cages and randomly assigned to experimental groups.
Blinding	The investigators were not blinded. The experiments were quantified and relevant appropriate statistical tests were performed.

Reporting for specific materials, systems and methods

We require information from authors about some types of materials, experimental systems and methods used in many studies. Here, indicate whether each material, system or method listed is relevant to your study. If you are not sure if a list item applies to your research, read the appropriate section before selecting a response.

Materials & experimental systems

n/a	Involved in the study	
☐	☒	Antibodies
☐	☒	Eukaryotic cell lines
☒	☐	Palaeontology and archaeology
☐	☒	Animals and other organisms
☒	☐	Clinical data
☒	☐	Dual use research of concern
☒	☐	Plants

Methods

n/a	Involved in the study	
☒	☐	ChIP-seq
☒	☐	Flow cytometry
☒	☐	MRI-based neuroimaging

Antibodies

Antibodies used	Antibody source was described in Materials and Methods section. The following antibodies and working concentrations were used

Antibodies used	for western blot: anti-HSP90 (1:10000, Santa Cruz Biotechnology sc-13119), anti-vinculin (1:10000, Sigma-Aldrich, v4505); anti-p53 DO-1 (1:1000 or 1:10000, Santa Cruz Biotechnology, sc-126); anti-LAT1/SLC7A5 (1:2000, Abcam, ab208776); anti-CD98hc/SLC3A2 (1:2000, Sigma-Aldrich, HPA017980); anti-ASCT2/SLC1A5 (1:2000, Cell Signaling Technology, #8057); anti-PSAT1 (1:2000, Proteintech, 10501-1-AP); anti-eIF2α D7D3 (1:5000, Cell Signaling Technology, #5324); anti-eIF2α phopsho S51 (1:1000, Cell Signaling Technology, #3398); anti-S6RP (1:1000, Cell Signaling Technology, #2317); anti-S6RP phospho S240/244 (1:1000, Cell Signaling Technology, #2215); anti-LC3 A/B (1:1000, Cell Signaling Technology, #12741); anti-HA (1:1000, Roche 1186742300); anti cleaved-Caspase 3 (1:1000, Cell Signaling Technology, #9664).<br>The following antibodies and working concentrations were used for immunofluorescence analysis:<br>anti-p53 Valentino (1:100, homemade); anti-γH2AX S139 (1:100, Millipore 05-636); anti-4EBP1 phospho T37/46 (1: 100, Cell Signaling Technology, #2855); anti-HA (1:100, Roche 1186742300).<br>The following primary antibodies and working concentrations were used for immunohistochemical stainings: anti-p53 (1:50 pH6, Leica Novocastra, NCL-L-p53-DO7), anti-LAT1/SLC7A5 (1:500 pH9, Abcam, ab208776), anti-PSAT1 (1:400 pH6, Proteintech, 10501-1-AP), anti-CD98hc/SLC3A2 (1:2500 pH9, Sigma Aldrich, HPA017980), anti-phospho-4EBP1 (1:1000 pH9, Cell Signaling, #2855); anti-γH2AX S139 (1:300, Millipore 05-636). Collagen fibers were stained with Picrosirius red stain (Bio Optica, 04-121873) as manufactuter's instructions.
Validation	Positive and/or negative controls such as protein knockdown, knockout, overexpression were previously used to validate the relevant antibodies used in this work.

Eukaryotic cell lines

Policy information about cell lines and Sex and Gender in Research

Cell line source(s)	ATCC or other laboratories cooperating on the project.
Authentication	Cells were subjected to STR genotyping with PowerPlex 18D System and confirmed in their identity comparing the results to reference cell databases (DMSZ, ATCC and JCRB databases).
Mycoplasma contamination	Cells were tested for mycoplasma contamination with negative results.
Commonly misidentified lines (See ICLAC register)	No commonly misidentified cell lines were used.

Animals and other research organisms

Policy information about studies involving animals; ARRIVE guidelines recommended for reporting animal research, and Sex and Gender in Research

Laboratory animals	See Materials and Methods section.
Wild animals	Study did not involve wild animals.
Reporting on sex	Only female BALB/c mice were injected with cells orthotopically into the mammary fat pad.
Field-collected samples	Study did not involve samples collected from the field.
Ethics oversight	Experimental protocol (Authorization n. 347/2022-PR) was approved by the Ethical Committee of the ICGEB and by the Italian Ministry of Health.

Note that full information on the approval of the study protocol must also be provided in the manuscript.
